# Editorial: Integrated PK/PD and drug metabolism approaches in drug development and evaluation

**DOI:** 10.3389/fphar.2026.1941847

**Published:** 2026-08-18

**Authors:** Fenglei Huang, Yurong Lai, André Dallmann, Xinning Yang

**Affiliations:** 1 Boehringer Ingelheim Pharmaceuticals, Inc., Ridgefield, CT, United States; 2 Drug Metabolism, Gilead Sciences Inc., Foster City, CA, United States; 3 Model-Informed Drug Development, Bayer Healthcare SAS, Lille, France; 4 US Food and Drug Administration, Silver Spring, MD, United States

**Keywords:** AI, drug metabolism, modeling, PBPK, pharmacodynamics, pharmacokinetics, QSP

## 1 Introduction

Pharmacokinetics (PK), pharmacodynamics (PD), and drug metabolism are fundamental to modern drug development, providing the quantitative basis for understanding drug exposure, efficacy, safety, and interindividual variability. Increasingly, integrated approaches that combine experimental pharmacology, bioanalysis, mechanistic modeling, and computational methods are transforming drug development by connecting evidence across discovery, translational research, clinical development, and precision medicine. These integrated strategies form the foundation of model-informed drug development (MIDD), supporting candidate selection, dose optimization, exposure–response characterization, therapeutic drug monitoring, and long-term safety assessment.

This Research Topic brings together nineteen contributions that illustrate how the integration of PK/PD and drug metabolism with physiologically based pharmacokinetic (PBPK) modeling, quantitative systems pharmacology (QSP), population PK/PD, artificial intelligence (AI), machine learning (ML), and bioanalytical sciences is advancing modern drug development. Together, these studies demonstrate how complementary quantitative approaches improve translational prediction, optimize dosing strategies, and support precision medicine. Rather than reviewing each article individually, this editorial highlights four major scientific themes emerging from the Research Topic and discusses their collective contributions to integrated, model-informed drug development.

## 2 Mechanistic modeling and model-informed drug development

Mechanistic modeling remains a cornerstone of model-informed drug development (MIDD), enabling quantitative integration of biological mechanisms with pharmacokinetic and pharmacodynamic knowledge to support drug discovery, translational research, dose optimization, and precision medicine. The four studies in this Research Topic illustrate how mechanistic modeling is being applied across the entire drug development continuum, from early lead optimization to clinical dose selection in special populations.

Xu et al. developed a mechanistic quantitative systems pharmacology (QSP) platform integrating multiple immune checkpoint pathways with extensive preclinical datasets to evaluate bispecific antibody target combinations, characterize dose–response relationships, and explore alternative dosing strategies. Model simulations further suggested that combining checkpoint-targeting bispecific antibodies with monoclonal antibodies could enhance antitumor efficacy, demonstrating the value of QSP for optimizing antibody design and translational development.

Expanding mechanistic modeling into early drug discovery, Zhang et al. integrated artificial intelligence with physiologically based pharmacokinetic (AI-PBPK) modeling to predict the pharmacokinetic and pharmacodynamic properties of aldosterone synthase inhibitors directly from their chemical structures. The framework enabled rapid prediction of PK/PD properties for multiple compounds and provides an efficient approach for lead optimization and candidate selection before clinical evaluation.

Using another complementary PBPK strategy, Zhang et al. combined quantitative structure–activity relationship (QSAR) modeling with mechanistic PBPK modeling to predict the human pharmacokinetics and tissue distribution of 34 fentanyl analogs. Besides improving prediction accuracy without extensive experimental data, the framework identified analogs with greater predicted central nervous system penetration, providing valuable insights into their potential abuse risk and supporting public health and regulatory risk assessment.

Moving from discovery to clinical development, Pokladyuk et al. established a PBPK model to characterize niraparib pharmacokinetics in patients with hepatic impairment. By incorporating disease-related physiological changes into the model, simulations successfully supported dose adjustments that maintained drug exposure within the desired therapeutic range, illustrating the value of mechanistic PBPK modeling for precision dosing in special populations.

Together, these studies illustrate how mechanistic modeling has evolved from describing pharmacokinetics to becoming an integrated decision-making framework that supports drug discovery, translational research, precision dosing, and clinical development.

## 3 Artificial intelligence and machine learning

Artificial intelligence (AI) and machine learning (ML) are increasingly complementing traditional pharmacometric and mechanistic modeling by improving prediction, integrating diverse datasets, and supporting data-driven decision-making throughout drug development. Rather than replacing mechanistic approaches, AI enhances their predictive capability and broadens their applications across drug discovery, precision dosing, and translational research.

At the drug discovery stage, Zhang et al. demonstrated how AI can be integrated with physiologically based pharmacokinetic (AI-PBPK) modeling to predict the pharmacokinetic and pharmacodynamic properties of aldosterone synthase inhibitors directly from their chemical structures. By enabling rapid screening and optimization of lead compounds before clinical evaluation, this framework illustrates the potential of AI to accelerate candidate selection while complementing mechanistic modeling.

The application of AI also extends into clinical practice. Jian et al. developed a machine learning-based model to predict teicoplanin plasma concentrations in patients with liver disease using real-world therapeutic drug monitoring data. Among ten evaluated algorithms, the LightGBM model achieved the best predictive performance by integrating clinical and laboratory variables, demonstrating how machine learning can improve individualized dosing and therapeutic drug monitoring in patients with altered pharmacokinetics.

Providing a broader perspective, Baran et al., in the only review article of this Research Topic, discussed the emerging role of hybrid mechanistic–machine learning PK/PD models that integrate digital biomarkers, continuous monitoring data, imaging, and multi-omics datasets. The authors highlight that AI is most powerful when combined with mechanistic pharmacology, improving pharmacokinetic prediction, covariate identification, and cross-species translation while emphasizing the importance of rigorous validation, interpretability, and regulatory acceptance.

Taken together, these advances suggest that AI will be most impactful when integrated with mechanistic pharmacology rather than used as a standalone predictive technology. By combining machine learning with mechanistic models and real-world clinical data, AI has the potential to accelerate drug discovery, optimize precision dosing, and support model-informed decision-making while maintaining biological interpretability and regulatory credibility.

## 4 Population PK/PD and precision medicine

Population PK/PD modeling remains a cornerstone of individualized therapy and an essential component of model-informed drug development. By quantifying interindividual variability and identifying clinically relevant covariates, these approaches support dose optimization, precision medicine, and evidence-based treatment across diverse patient populations. The studies in this Research Topic demonstrate the expanding role of population PK/PD modeling in pharmacogenomics, biosimilar development, special populations, precision dosing, and long-term safety evaluation.

The integration of population PK modeling with pharmacogenomics is illustrated by Wang et al., who investigated the influence of ABCB1 polymorphisms on rivaroxaban exposure and clinical outcomes in Chinese patients with non-valvular atrial fibrillation. The study linked genetic variants to differences in drug exposure, bleeding risk, and thromboembolic events, highlighting the potential of combining pharmacogenetics with population PK modeling to support individualized anticoagulant therapy.

Population PK/PD modeling also continues to play an important role in clinical development and regulatory evaluation. Choi et al. demonstrated the biosimilarity of SB16 to reference denosumab using an integrated population PK/PD model, showing comparable pharmacokinetic profiles, pharmacodynamic responses, and clinical outcomes while confirming that demographic covariates had no clinically meaningful impact on treatment response. In a second study, Choi et al. characterized the population pharmacokinetics of efpeglenatide using pooled data from six clinical studies in obesity and type 2 diabetes. Although body weight and disease status influenced pharmacokinetic parameters, model-based simulations supported a uniform dosing strategy and stepwise dose escalation, providing quantitative evidence to support clinical development.

Several studies further demonstrated the value of population PK/PD modeling in optimizing therapy for special populations. He et al. developed a PopPK/PD model linking YPEG-rhGH exposure to insulin-like growth factor-1 (IGF-1) responses in elderly subjects, identifying age and body weight as important determinants of pharmacokinetic variability and supporting flexible dosing strategies. Similarly, Zhu et al. integrated pharmacokinetic, pharmacodynamic, and simulation analyses to optimize continuous infusion regimens of ciprofol in elderly surgical patients, identifying dosing strategies capable of maintaining the desired depth of anesthesia while improving individualized perioperative care.

Population modeling also provides a quantitative framework for balancing efficacy, safety, and long-term risk management. Wu et al. developed a population PK model to optimize linezolid infusion strategies in critically ill patients, demonstrating through Monte Carlo simulations that prolonged intermittent infusion improved target attainment whereas continuous infusion could increase toxicity, particularly for pathogens with higher minimum inhibitory concentrations. Complementing this work, Hanke et al. established a population PK/PD model describing the relationship between long-term iclepertin exposure and hemoglobin levels in patients with schizophrenia. Model simulations predicted only small and reversible reductions in hemoglobin, providing quantitative support for long-term safety assessment and precision dosing in vulnerable patient populations.

The breadth of applications represented here underscores how population PK/PD modeling has evolved from describing variability to supporting precision medicine across the entire clinical development pathway. By integrating patient characteristics, genetics, exposure–response relationships, and clinical outcomes, these approaches increasingly support individualized therapy, regulatory evaluation, and model-informed clinical development across diverse therapeutic areas.

## 5 Clinical pharmacology, therapeutic drug monitoring and bioanalytical sciences

Clinical pharmacology, bioanalytical sciences, and therapeutic drug monitoring provide the experimental foundation for model-informed drug development. Together, these complementary disciplines generate high-quality pharmacokinetic and pharmacodynamic data needed to characterize drug disposition, optimize dosing strategies, and translate quantitative models into clinical practice.

Advances in bioanalytical technologies continue to improve the characterization of drug metabolism and disposition. Xu et al. developed and validated a sensitive UHPLC–MS/MS method to systematically characterize the pharmacokinetics and metabolism of the marine-derived indole alkaloid meridianin C in rats. The study identified the major metabolic pathways and metabolites while demonstrating that meridianin C remains the predominant circulating compound following oral administration, providing the first comprehensive pharmacokinetic evaluation of this promising therapeutic candidate. Complementing this work, Gao et al. developed and validated a rapid two-dimensional liquid chromatography (2D-LC) method for therapeutic drug monitoring of pyrotinib in human plasma. The assay demonstrated excellent analytical performance and successfully characterized substantial interindividual pharmacokinetic variability in patients with HER2-positive breast cancer, providing a practical tool to support individualized dosing in precision oncology.

Several studies further demonstrated how experimental pharmacology and therapeutic drug monitoring can improve translational research and clinical decision-making. Zhao et al. investigated the cerebrospinal fluid pharmacokinetics and PK/PD profiles of meropenem, vancomycin, and tigecycline in patients with central nervous system infections following neurosurgery. The marked variability in antibiotic exposure highlights the value of PK/PD-guided therapeutic drug monitoring for optimizing antimicrobial therapy in this challenging clinical setting. In a complementary preclinical study, Liu et al. showed that intravenous bolus injection rate substantially influences the pharmacokinetics, pharmacodynamics, and safety of anesthetic agents in rats. Faster administration produced higher peak drug concentrations, more rapid onset of anesthesia, prolonged pharmacodynamic effects, and increased adverse events, emphasizing that administration strategy itself is an important experimental variable in translational pharmacology.

Clinical pharmacology studies also continue to provide essential evidence for dose selection and clinical development. Liu et al. characterized the pharmacokinetics, pharmacodynamics, and safety of tolvaptan following single- and multiple-dose administration in healthy Chinese volunteers, demonstrating predictable exposure, dose-proportional pharmacokinetics, and good tolerability that support future clinical development. Similarly, Yang et al. evaluated the novel intravenous anesthetic ET-26 in healthy elderly and non-elderly subjects. Although systemic exposure was higher in elderly participants, pharmacodynamic responses and overall tolerability remained comparable between age groups, indicating that routine dose adjustment is unnecessary while supporting the continued clinical development of ET-26 as a potentially safer alternative to etomidate.

These studies reinforce that advances in quantitative pharmacology depend not only on increasingly sophisticated computational models but also on robust bioanalytical methods, therapeutic drug monitoring, and carefully designed clinical pharmacology studies. Together, they provide the experimental evidence that validates model-informed approaches and enables their translation into safer and more effective patient care.

## 6 Conclusion and future perspectives

This Research Topic highlights the breadth and maturity of modern quantitative clinical pharmacology. The nineteen contributions span the entire drug development continuum—from computational drug discovery, preclinical experimentation, and first-in-human studies to biosimilarity assessment, therapeutic drug monitoring, precision dosing, and long-term safety evaluation. Despite covering diverse therapeutic areas, all studies demonstrate how quantitative pharmacology improves decision-making throughout drug development.

Collectively, these articles demonstrate that integrated quantitative pharmacology has become indispensable for modern drug development. Beyond their individual scientific contributions, they reveal several emerging trends, including the increasing integration of mechanistic and data-driven modeling, wider adoption of artificial intelligence and real-world evidence, expansion of population PK/PD into pharmacogenomics and long-term safety assessment, continued advances in bioanalytical sciences and therapeutic drug monitoring, and broader implementation of model-informed drug development across the product lifecycle.

Looking ahead, quantitative clinical pharmacology is evolving beyond predicting drug exposure and selecting doses. Mechanistic modeling, PBPK, QSP, population PK/PD, artificial intelligence, machine learning, digital biomarkers, and real-world evidence are increasingly being integrated into unified decision-making frameworks that support precision medicine. Continued collaboration among pharmacologists, clinicians, modelers, statisticians, bioanalytical scientists, and AI researchers will be essential to accelerate the development of safer, more effective, and increasingly personalized medicines.

